# Supplementary material for: Modelling the evolution of transcription factor binding preferences in complex eukaryotes
Source: Sci Rep. 2017 Aug 8;7:7596. doi: 10.1038/s41598-017-07761-0 (PMC5548724; doi:10.1038/s41598-017-07761-0)
Supplement: Supplementary file 1 — Supplementary Information [file 41598_2017_7761_MOESM1_ESM.pdf]

Supplementary Information

Modelling the evolution of transcription factor binding preferences in complex eukaryotes

Antonio Rosanova [1], Alberto Colliva [1], Matteo Osella [1],Michele Caselle [1]

[1]Department of Physics and INFN, Università degli Studi di Torino, via P.Giuria 1, I-10125 Turin, Italy

Contents

1 Maximum Likelihood Estimation (MLE) 2

2 $M \to \infty$ is a good approximation 3

3 Size distribution of motif families for Zinc Finger and Homeobox TFs 5

4 θ does not depend on the number of TFs. 6

5 Three illustrative examples of the splitting of DBD families in motif families 7

5.1 The {IRF} family 7

5.2 The {IRX} family 7

5.3 The {HOX} family 7

6 Robustness of the motif family organization 10

6.1 PWM similarity as an alternative way to group transcription factor binding preferences 10

6.2 A TF-TF network expansion based on PWM similarity does not significantly alter the motif family organization 10

7 Evolutionary origin of isolated TFs 14

8 List of connected components of the motif familes network 15

1 Maximum Likelihood Estimation (MLE)

In this section we derive the maximum likelihood estimator for the parameter θ of the discrete logarithmic distribution. The properly normalized discrete logarithmic distribution, for $k \geq 1$ and $0 < \theta < 1$, is given by:

$$p(k|\theta) = \frac{-1}{\ln(1-\theta)} \frac{\theta^k}{k} \tag{1}$$

Given the data sample $\{k_j\}_{j=1,...F}$, where k_j is the size of j-th family and F the total number of families, the likelihood is defined as:

$$\mathscr{L}(\theta|\{k_j\}) = \prod_{j=1}^{F} p(k_j|\theta) = \left[\frac{-1}{\ln(1-\theta)}\right]^F \prod_{j=1}^{F} \frac{\theta^{k_j}}{k_j}$$

As usual, we will treat the log-likelihood:

$$\begin{aligned} \ln \mathscr{L}(\theta|\{k_j\}) &= -F \ln\left(-\ln(1-\theta)\right) + \ln\theta \sum_{j=1}^{F} k_j - \sum_{j=1}^{F} \ln(k_j) \qquad (2) \\ &= -F \ln\left(-\ln(1-\theta)\right) + F\overline{k} \ln\theta - F \ln M_g \qquad (3) \end{aligned}$$

where $\overline{k}$ is the arithmetic mean over the sample and M_g is the geometric mean over the sample. To maximize the log-likelihood:

$$\left.\frac{\partial \ln \mathscr{L}}{\partial \theta}\right|_{\theta=\theta_{MLE}} = 0 \implies \theta_{MLE} + \overline{k}(1-\theta_{MLE})\ln(1-\theta_{MLE}) = 0$$

So the ML estimator θ_{MLE} depends only on the arithmetic mean over the sample. To obtain the explicit form of θ_{MLE} we rewrite the equation in terms of $t = ln(1-\theta_{MLE})$ and take advantage of the Lambert W Function defined by $z = W(z)e^{W(z)}$ for any complex number z. Then

$$\begin{aligned} \theta_{MLE} + \overline{k}(1-\theta_{MLE})\ln(1-\theta_{MLE}) = 0 &\implies 1 - e^t + \overline{k}\, t\, e^t = 0 \\ &\implies \left(t - \frac{1}{\overline{k}}\right) e^t = -\frac{1}{\overline{k}} \implies \left(t - \frac{1}{\overline{k}}\right) e^{t-\frac{1}{\overline{k}}} = -\frac{1}{\overline{k}} e^{-\frac{1}{\overline{k}}} \\ &\implies t - \frac{1}{\overline{k}} = W\left(-\frac{1}{\overline{k}} e^{-\frac{1}{\overline{k}}}\right) \implies \theta_{MLE} = 1 - e^{\frac{1}{\overline{k}} + W\left(-\frac{1}{\overline{k}} e^{-\frac{1}{\overline{k}}}\right)} \end{aligned}$$

Given that $\overline{k} \geq 1$, we have $-\frac{1}{e} \leq -\frac{1}{\overline{k}} e^{-\frac{1}{\overline{k}}} < 0$. In this range of values the Lambert W function is well defined and double-valued. The two solutions fulfill different functional properties. Since $-\frac{1}{\overline{k}} \geq -1$, the branch 0 solution fulfills the following property: $W_0(ze^z) = z$. thus in this case

$$\theta_{MLE} = 1 - e^{\frac{1}{\overline{k}} + W_0\left(-\frac{1}{\overline{k}} e^{-\frac{1}{\overline{k}}}\right)} = 0$$

leading to un unphysical solution.

On the contrary, for the branch -1 solution we have $-\infty < W_{-1}(z) < -1$, which gives an acceptable solution with $0 \leq \theta_{MLE} < 1$.

$$\theta_{MLE} = 1 - e^{\frac{1}{\overline{k}} + W_{-1}\left(-\frac{1}{\overline{k}} e^{-\frac{1}{\overline{k}}}\right)} \geq 0$$

This is the solution we used to estimate the best fit values of θ for the various distribution studied in the main text.

2 $M \to \infty$ is a good approximation

An important step of the previous analysis was the $M \to \infty$ limit which allowed us to obtain a closed, analytic form for the probability distribution. We shall evaluate here the error which we may expect on the probability distribution due to this approximation.

We have shown that the number of families of size i is described by $f_i \sim \frac{\theta^i}{i}$. So the probability of having a family of size i is given by:

$$p_{i,M} = \frac{f_i}{\sum_{j=1}^{M} f_j} = \frac{\theta^i/i}{\sum_{j=1}^{M} \theta^j/j} \tag{4}$$

Where M is the maximal size for a family. In the above calculation we used instead the $M \to \infty$ expression:

$$p_{i,\infty} = \frac{\theta^i/i}{\sum_{j=1}^{\infty} \theta^j/j} = -\frac{1}{\ln(1-\theta)} \frac{\theta^i}{i} \tag{5}$$

We want to estimate the difference $p_{i,M} - p_{i,\infty}$ which is (obviously) a decreaseing function of M. Our goal is to find for any chosen ϵ the minimum value of M, which we shall call $M_{min}(\theta, \epsilon)$, such that for $M > M_{min}(\theta, \epsilon)$ the relative error is $\frac{p_{i,M} - p_{i,\infty}}{p_{i,M}} < \epsilon$

Let us define $F_\infty = \sum_{j=1}^{\infty} f_j = -\ln(1-\theta)$ and $F_M = \sum_{j=1}^{M} f_j = F_\infty - R$ where $R = \sum_{j=M+1}^{\infty} f_j$.

$$p_{i,M} - p_{i,\infty} = \frac{f_i}{F_M} - \frac{f_i}{F_\infty} = \frac{f_i}{F_M}\frac{R}{F_\infty} = p_{i,M}\frac{R}{F_\infty}$$

So the relative error is given by $\frac{p_{i,M} - p_{i,\infty}}{p_{i,M}} = \frac{R}{F_\infty}$. Given that $\theta^j/j < \theta^j/M$ for any $j > M$, we have that:

$$R = \sum_{j=M+1}^{\infty} \frac{\theta^j}{j} < \sum_{j=M+1}^{\infty} \frac{\theta^j}{M}$$

$$R < \frac{1}{M}\left[\sum_{j=0}^{\infty} \theta^j - \sum_{j=0}^{M+1} \theta^j\right]$$

$$R < \frac{1}{M}\left[\frac{1}{1-\theta} - \frac{1-\theta^{M+2}}{1-\theta}\right]$$

$$R < \frac{1}{M}\frac{\theta^{M+2}}{1-\theta}$$

Then:

$$\frac{p_{i,M} - p_{i,\infty}}{p_{i,M}} < -\frac{1}{M}\frac{\theta^{M+2}}{(1-\theta)\ln(1-\theta)}$$

Thus in order to have a relative error smaller than ϵ, it is enough to impose:

$$\epsilon = -\frac{1}{M}\frac{\theta^{M+2}}{(1-\theta)\ln(1-\theta)}$$

Which can be solved as follows:

$$
\begin{aligned}
-M\theta^{-M} &= \frac{1}{\epsilon}\frac{\theta^2}{(1-\theta)\ln(1-\theta)} \\
-M\ln\theta \quad e^{-M\ln\theta} &= \frac{1}{\epsilon}\frac{\theta^2}{(1-\theta)}\frac{\ln\theta}{\ln(1-\theta)} \\
-M\ln\theta = W\left(\frac{1}{\epsilon}\frac{\theta^2}{(1-\theta)}\frac{\ln\theta}{\ln(1-\theta)}\right)& \\
M = -\frac{1}{\ln\theta}W\left(\frac{1}{\epsilon}\frac{\theta^2}{(1-\theta)}\frac{\ln\theta}{\ln(1-\theta)}\right)&
\end{aligned}
$$

where $W(z)$ denotes, as above, the Lambert function. This is the minimum acceptable value of M for which the relative error is smaller than ϵ for a given θ. For example for $\epsilon = 0.01$ at $\theta = 0.74$ M needs to be greater than 10 a condition which is indeed fulfilled by our data, As θ decreases also the threshold M_{min} decreases. In the fouth cases discussed in the main text we find $M_{min} = 1$ for $\theta = 0.22$ (yeast), $M_{min} = 2$ for $\theta = 0.30$ (caenorhabditis), $M_{min} = 4$ for $\theta = 0.51$ (drosophila) and $M_{min} = 10$ for $\theta = 0.75$ (mouse). We checked that in all the above cases this condition is indeed fulfilled by the data.

3 Size distribution of motif families for Zinc Finger and Homeobox TFs

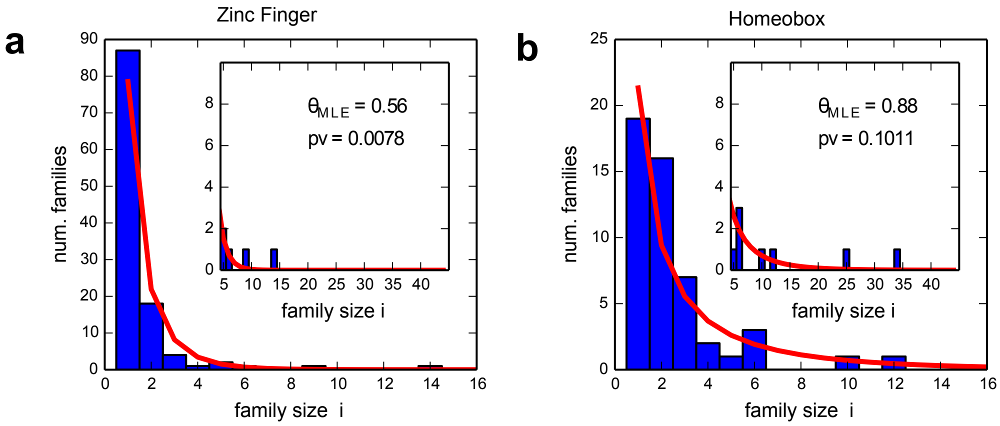

Supplementary Figure S1: **Size distribution of motif families for specific DBDs. a)** The distribution accounts for 178 human Zinc Finger TFs, organized in 115 families whose members share at least one PWM with at least another member. **b)** The distribution accounts for 184 human Homeobox TFs, organized in 52 motif families. Both the insets show a zoom on the range of sizes > 5. Red-lines represent the best-fit models according to maximum likelihood estimation.

4 θ does not depend on the number of TFs.

The figure shows that θ does not depends on the number of TFs, but only on how the TFs are arranged in families.

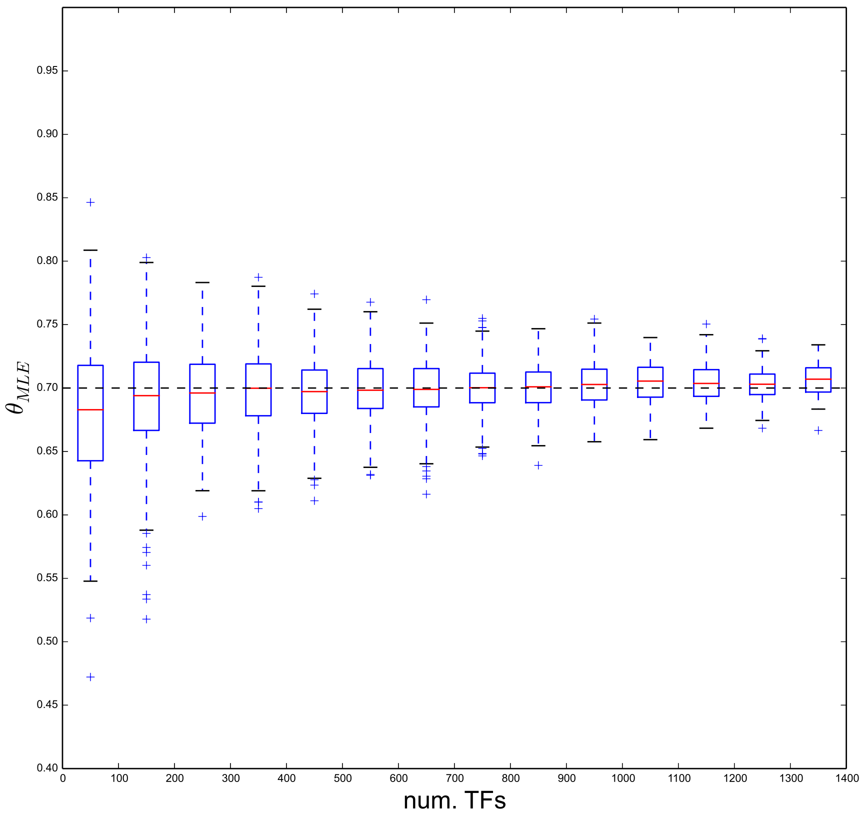

Supplementary Figure S2: **θ does not depend on the number of TFs.** We ran $5 * 10^3$ simulations of the model for different system sizes, corresponding to the different numbers of TFs. The rates of duplication, deletion and cis-innovation are set by $\theta = 0.7$ and by the additional constraint $\lambda = \delta$. Simulations are let to run for $5 * 10^4$ to the steady state and the θ_{MLE} is computed.

5 Three illustrative examples of the splitting of DBD families in motif families

This section discusses three prototypical examples of DBD families and how they split into motif families. More specifically these examples cover three possible scenarios: a DBD family that splits into a set of single copy transcription factors (thus potential singletons); a family in which the DBDs are so well conserved that all the components belong to the same motif family; and finally an intermediate situation in which the DBD family splits into several motif families of different sizes.

5.1 The {IRF} family

In human, the interferon regulatory transcription factor (IRF) family is composed by nine elements denoted as IRF1....IRF9. For each of them, except for IRF6, there are several *experimentally validated* PWMs. For instance, for IRF1 (which was the first member of the IRF family identified) there are 6 known PWMs: four are present in the TRANSFAC database [1], one in the HOCOMOCO database [2] and one in the JASPAR database [3]. These PWMs are very similar, although not identical, and are thus treated in the CIS-BP database as independent entries. The DBD domain of IRF1 has a strong homology with the DBDs of the orthologous IRF1 in other vertebrates, but low homology with any other human TF. Therefore, there are no PWMs from other TFs that can be additionally associated to IRF1 by inference. The same occurs for all the remaining IRFs, except for IRF6 for which there is no direct experimental evidence. There is however a PWM for the mouse version of IRF6 obtained using Protein Binding Microarrays and reported in ref. [4]. Thanks to the very high level of homology (100% identity of the two DBDs) between the human and mouse versions of IRF6, this PWM can be inferred to hold also for the human version of IRF6. In this way, we end up with at least one PWM for each IRF gene. Since there is no common PWM between any pair of IRFs, each of them is present as a singleton in our network. It is easy to see that this result is a consequence of the low level of homology between the DBDs of different human IRF TFs (so that no PWM of a given human IRF could be inferred to hold also for another human IRF) and thus correctly reproduces the fact that they substantially diverged during evolution. As a matter of fact, a direct inspection (Figure S4, panel a) immediately shows that the PWMs of different human IRFs are indeed very different among them, in contrast with the high similarity of PWMs obtained with different experimental methods for the same IRF, as mentioned above in the IRF1 case.

5.2 The {IRX} family

A radically different situation is that of the IRX family. The Iroquois homeobox factors (IRX) compose a family of homeodomain TFs that play a crucial role in many developmental processes [5]. In human, this family is composed by six elements denoted as IRX1...IRX6. Only for two of them: IRX2 and IRX5 there is an experimentally validated PWM, obtained, in both cases, using SELEX technology [6]. The PWMs of the other four can be inferred from these two, thanks to the high degree of homology among these TFs. More precisely the DBDs of IRX2 and IRX5 have a 100% level of homology between them (and indeed the two PWMs are very similar and almost coincide), IRX6 has a 90% homology with (IRX2, IRX6) and IRX1, IRX3, IRX4 (which in turn are very similar among them) have 88% level of homology with (IRX2, IRX6). As a final result all the 6 TFs share the same pair of PWMs and are thus joined together in the same motif family. In Figure S4b a typical PWM similarity within the group is shown.

5.3 The {HOX} family

As a final example let us study an intermediate situation. The HOX family is composed by a large number of TFs which turn out to split in three motif families in our network. There is a large motif family composed by 34 TFs out of which 31 comes from HOX genes, and two smaller families of 4 and 2 elements respectively. In particular, the family with 4 elements is composed by the {HOXA13, HOXB13, HOXC13, HOXD13} proteins. These are well studied and important TFs for which several PWMs have been characterized experimentally with a wide range of different techniques. More precisely:

- HOXA13 is associated in CIS-BP to 7 different *experimentally validated* PWMs. Two are taken from the Transfac database [1], four are the results of SELEX experiments [6] and the last one is taken from the HOCOMOCO database [2].

- HOXB13 is associated to three PWMs, one obtained with Protein Binding Microarrays [7], and the other two with Selex [6].
- HOXC13 is associated to only two PWMs, both from SELEX experiments [6].
- HOXD13 is associated to three PWMs, one is taken from the HOCOMOCO database [2] and the other two were obtained with Selex [6].

What is important for our analysis is that the DBDs of these four TFs show a very high level of homology among them (Figure S3).

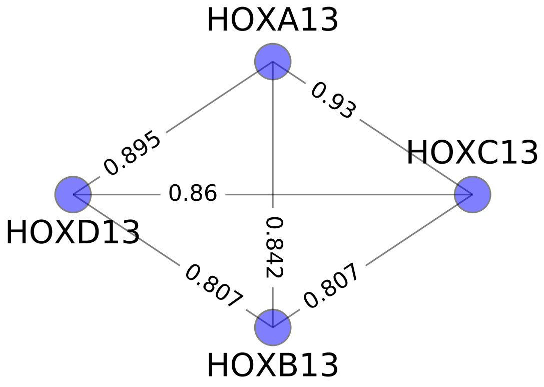

Supplementary Figure S3: **Homology of the DBDs of the four transcription factors HOXA13, HOXB13, HOXC13, HOXD13 composing a single motif family.** The degree of homology reported is obtained from the CIS-BP database [7]

Thus **all** the PWMs listed above can be inferred to hold also for **all** the remaining HOX*13 genes. On the contrary, these proteins show a rather low degree of homology in their DBDs (below 70%) with the remaining HOX. For this reason, no PWM corresponding to any other HOX TF can be associated to the HOX*13 TFs by inference. As a final result, these four HOX*13 TFs compose a single motif family, which is distinct from the main one containing almost all the remaining HOX TFs. Figure S4 show that indeed the PWM similarity among HOX*13 TF is really high (panel c) with respect to the negligible similarity of PWMs of elements of HOX*13 with other HOX TFs (panel d).

This last example allows us to discuss another important issue related to the inference protocol of the CIS-BP database. Each DBD within the CIS-BP database is characterized by a different threshold which separates high-homology TFs (from which the PWMs can be inferred) from low-homology ones. In the HOX case discussed here, the threshold is located at 70% of homology, but it can be different for different DBDs depending on their specific physical properties.

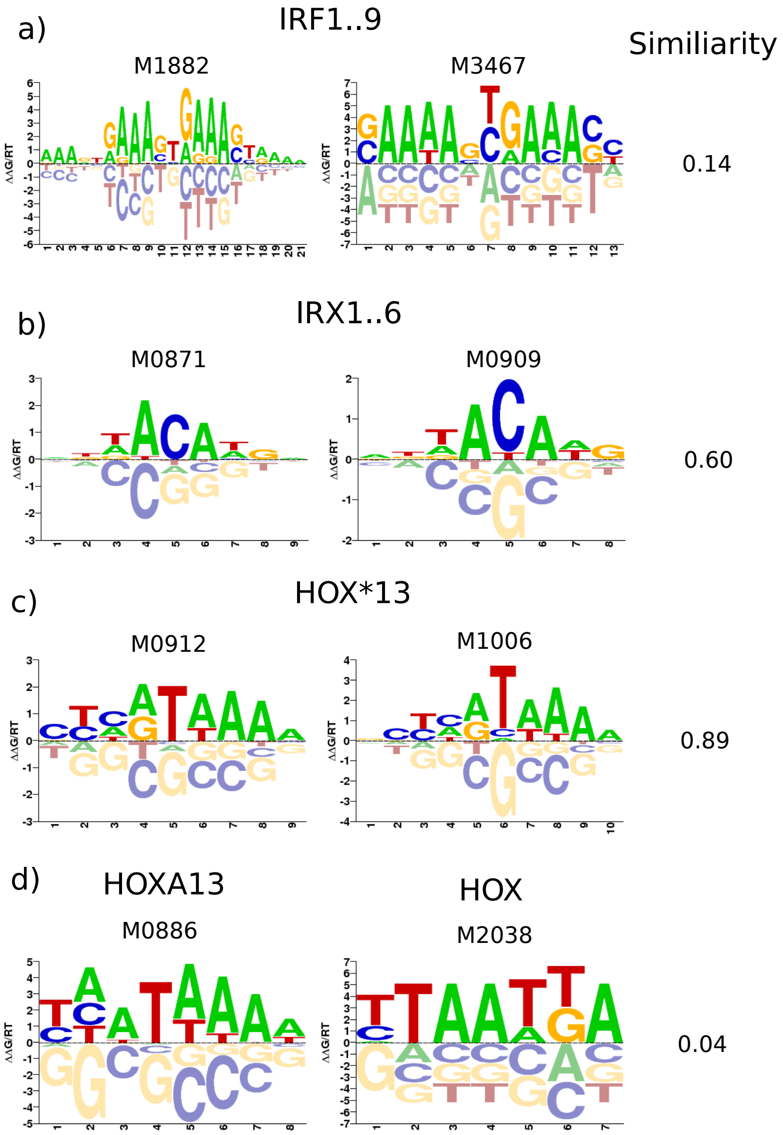

Supplementary Figure S4: **The PWM similarities measured with the Jaccard index for pairs of TFs coherently reflect their motif family associations**. The similarity of PWMs of TFs belonging to the same DBD family of IRF1..9 is extremely low (panel a) and indeed they are all single copy TFs in the motif family organization. On the other hand, the IRX1..6 family (panel b) does not split into independent motif families and coherently the associated PWMs have a high degree of similarity. Finally, motifs associated to the motif family of HOX*13 TFs have a strikingly high value of in-group similarity (panel c), as opposed to a very low value when compared to motifs associated to other HOX member that still belong to the same DBD family but are associated to a different motif family by our procedure.

6 Robustness of the motif family organization

Our definition of motif families is based on the CIS-BP database (version number 1.02), which in turn is based on a combination of experimental evidence and DBD homology. In order to assess the robustness of our construction, this section studies the changes induced in the TF-TF network, and consequently in the composition of its connected components (i.e., the motif families), if a different and less stringent definition of links is adopted. So far, we considered the TF-TF network in which a link is present if two TFs share at least one identical PWM according to the CIS-BP database. This choice could in principle generate errors in the network construction if two very similar PWMs are actually annotated as different in the data source. To test this possible issue, we introduce a measure of similarity between PWMs and thus expand the TF-TF network using different thresholds for this similarity measure to define new potential links.

6.1 PWM similarity as an alternative way to group transcription factor binding preferences

We downloaded all PWMs and their associations to TFs from CIS-BP (version 1.02). To avoid the noise due to PWMs with low information content (IC), we filtered out the matrices with IC less than 10 bits. We measured the IC as the Kullback–Leibler (KL) distance between the motif and the overall genome composition

$$I = \sum_{i=1}^{L} \sum_{b \in \{A,C,T,G\}} p_i(b) \log_2(p_i(b)/q(b)),$$

where L is the length of the motif, $p_i(b)$ is the frequency of base b at position i in the motif and $q(b)$ is its background frequency. After this filtering step, we performed a similarity analysis between pairs of PWMs using the Jaccard index as in ref. [8]. In this context, the Jaccard index measures the fraction of sequences recognized by a pair of PWMs in the larger set of sequences recognized by any of the two. A completely connected weighted network of all PWMs with $IC > 10$ bits can now be built with weights defined by this similarity measure. The distribution of this weights turned out to be roughly exponential.

6.2 A TF-TF network expansion based on PWM similarity does not significantly alter the motif family organization

The TF-PWM network defined by the associations in the CIS-BP database is now coupled to the weighted PWM-PWM network defined above. A threshold can now be defined on the PWM similarity measure to prune the PWM-PWM network and keep only the connections corresponding to a sufficiently high similarity. For any given value of the threshold, there is a natural way to define the TF projected network that we used in the main text. This is a TF-TF network in which a link is present between two TFs if there is at least one connected path joining them through their associated motifs in the combined TF-PWM network. Once again, the network connected components define the motif families. This procedure is sketched in Figure S5.

With this procedure, the motif family organization in principle depends on the choice of the threshold for PWM similarity. Two extreme cases can be easily defined:

- a threshold value close to zero conserves all the links between PWMs, thus defining a fully connected network of TFs;

- if a perfect identity between PWMs is required to cross the threshold, we recover the procedure described in the main text, in which a link between two TFs is present only if they have at least one motif in common.

Exploring threshold values in between these two extreme scenarios opens the possibility to test the robustness of the motif family organization as a function of the similarity threshold between PWMs. In other words, we can check how much our definition of the motif family structure is resilient to the addition of new links in the underlying PWM network and thus indirectly how much can be affected by possible annotation errors in the original database. Note that the threshold value corresponds to the level of similarity considered sufficient to state that two TFs have essentially the same binding preferences, and thus potentially the same set of target genes.

Supplementary Figure S6a reports the number of links that are added to the projected network between TFs as a function of the threshold value for PWM similarity (measured with the Jaccard index), and of the corresponding

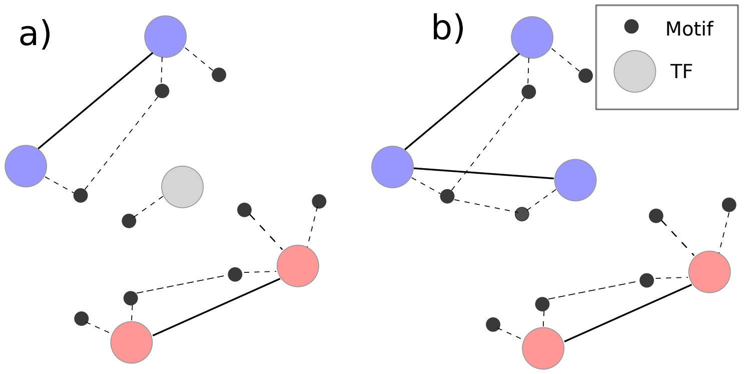

Supplementary Figure S5: **Sketch of TF-TF network construction.** Links between PWMs (dashed lines) are present if their similarity is above a defined threshold value. In the projected network between TFs, a link (solid line) is defined if there is path between motifs (i.e., a connected dashed path) that connects the two TFs .

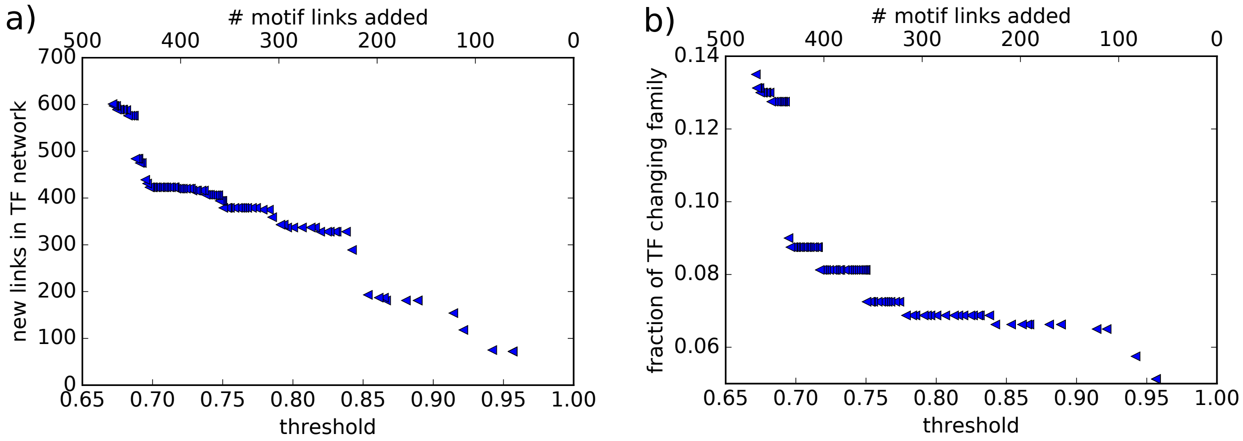

Supplementary Figure S6: **Robustness of the motif family organization as the network is expanded using similarity between PWMs.** a) The panel shows the approximately linear increase of the number of links added to the projected TF-TF network as a function of the similarity threshold value chosen to define the links between PWMs (bottom x-axis) and of the corresponding number of new links between PWMs (top x-axis). b) The corresponding fraction of TFs that change their motif family due to the lowering of the similarity required between PWMs is reported. Changes in the motif family organization are evaluated with respect to the motif family structure defined by a similarity threshold of 1, which corresponds to the organization discussed in the main text. Essentially, if a new added link between two PWMs induces a merging of a motif family of size N_1 and a motif family of size N_2, we consider that $N_1 + N_2$ TFs have changed their motif family association.

added links between PWMs. Even if this absolute number can be substantial, it does not correspond to a significant reorganization of the motif family organization. This is shown in Figure S6b: the fraction of TFs that change motif family due to this network expansion is very low for a wide range of threshold values. For example, this fraction is still below 10% for a threshold value of 0.7 which corresponds to more than 400 new potential links in the TF network. In fact, the number of new links in the TF network increases almost linearly with the threshold (with bumps which may be related to the modular structure of the network), but most of them coincide with already existing links between TFs or with links that do not merge different motif families. This is clearly illustrated by three examples reported in Figure S7.

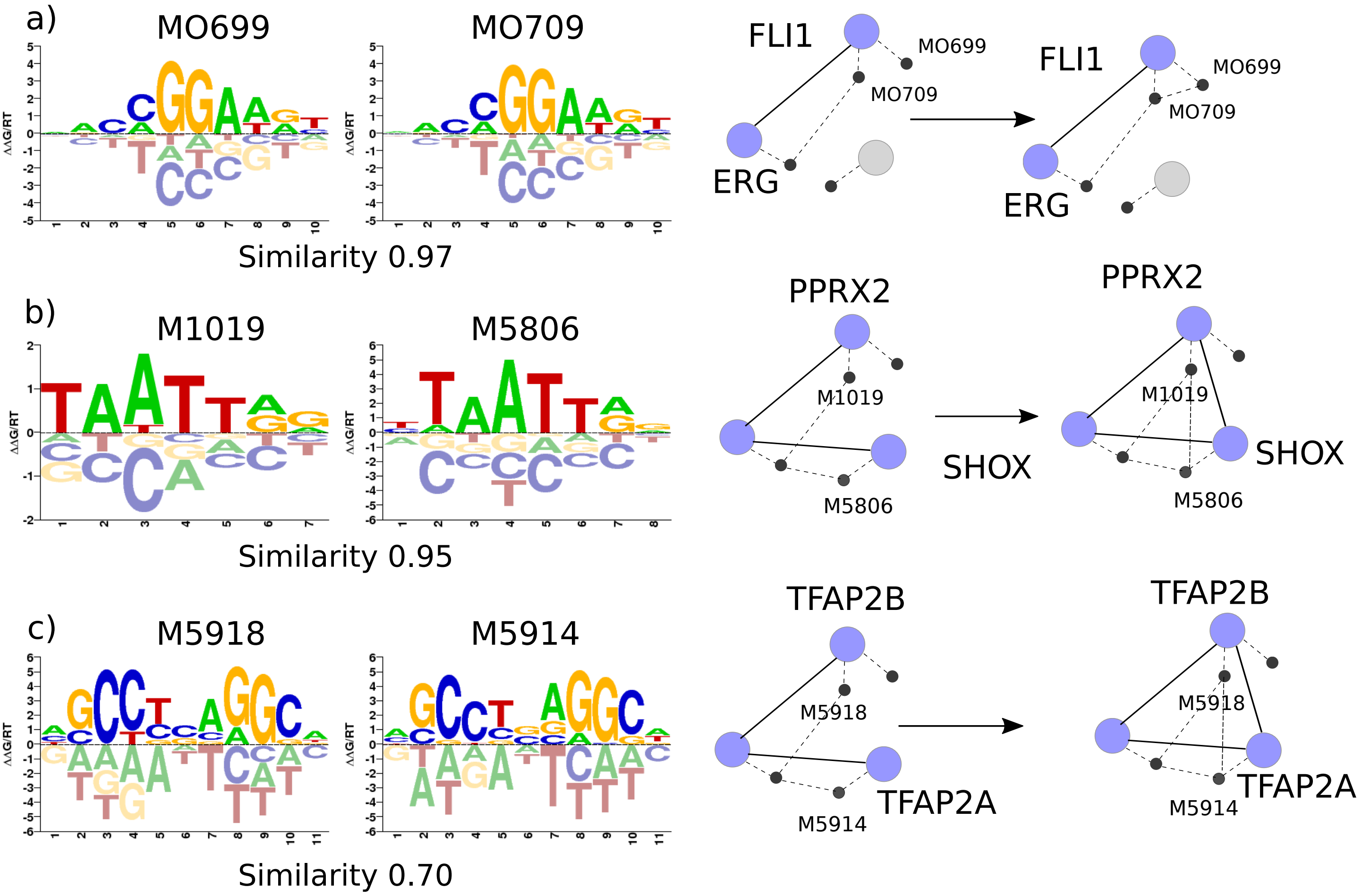

Supplementary Figure S7: **Typical examples of added links between similar PWMs that do not change the motif family structure**. For decreasing values (from panel a to panel c) of the similarity threshold, measured with the Jaccard index, new links are added to the PWM network. However, often these links connect TFs that are already connected (panel a) or that are in the same connected component (panel b and c). Therefore, they do not affect the motif family organization. The identifiers on top of each motif logo correspond their CIS-BP IDs.

References

[1] Matys, V. *et al.* Transfac® and its module transcompel®: transcriptional gene regulation in eukaryotes. *Nucleic Acids Res.* **34**, D108–D110 (2006).

[2] Kulakovskiy, I. V. *et al.* Hocomoco: a comprehensive collection of human transcription factor binding sites models. *Nucleic acids research* **41**, D195–D202 (2012).

[3] Mathelier, A. *et al.* Jaspar 2014: an extensively expanded and updated open-access database of transcription factor binding profiles. *Nucleic acids research* gkt997 (2013).

[4] Badis, G. *et al.* Diversity and complexity in dna recognition by transcription factors. *Science* **324**, 1720–1723 (2009).

[5] Gómez-Skarmeta, J. L. & Modolell, J. Iroquois genes: genomic organization and function in vertebrate neural development. *Current opinion in genetics & development* **12**, 403–408 (2002).

[6] Jolma, A. *et al.* Dna-binding specificities of human transcription factors. *Cell* **152**, 327–339 (2013).

[7] Weirauch, M. T. *et al.* Determination and inference of eukaryotic transcription factor sequence specificity. *Cell* **158**, 1431–1443 (2014).

[8] Vorontsov, I. E., Kulakovskiy, I. V. & Makeev, V. J. Jaccard index based similarity measure to compare transcription factor binding site models. *Algorithms for Molecular Biology* **8**, 23 (2013).

7 Evolutionary origin of isolated TFs

TF	DBD	Last Common Ancestor	TF	DBD	Last Common Ancestor
BRF1	UNKNOWN	LUCA	SIN3A	UNKNOWN	Eukarya
GABPB1	UNKNOWN	LUCA	LTF	UNKNOWN	Eukarya
ENO1	UNKNOWN	LUCA	NFYC	UNKNOWN	Eukarya
PURA	UNKNOWN	LUCA	TOPORS	UNKNOWN	Eukarya
HDAC2	UNKNOWN	LUCA	RAD21	UNKNOWN	Eukarya
NFYB	UNKNOWN	LUCA	NROB1	UNKNOWN	Eukarya
BCL3	UNKNOWN	LUCA	MYRF	NDT80_PhoG	Eukarya
CHD2	UNKNOWN	LUCA	ING4	GAGA_bind	Eukarya
SIRT6	UNKNOWN	LUCA	MTERF1	mTERF	Eukarya
HLTF	UNKNOWN	LUCA	NFYA	CBFB_NFYA	Eukarya
CTNNB1	UNKNOWN	LUCA	BRCA1	EIN3	Eukarya
GTF2B	UNKNOWN	LUCA	CENPB	CENP-B_N	Eukarya
SMC3	UNKNOWN	LUCA	LIN54	CXC	Eukarya
ZNF350	UNKNOWN	LUCA	EBF4	UNKNOWN	Opisthokonta
POLR3A	UNKNOWN	LUCA	EBF3	UNKNOWN	Opisthokonta
HDAC1	UNKNOWN	LUCA	EBF2	UNKNOWN	Opisthokonta
TAF1	UNKNOWN	Eukarya	RBPJ	LAG1-DNAbind	Opisthokonta
BPTF	UNKNOWN	Eukarya	NRF1	UNKNOWN	Metazoa
LMO2	UNKNOWN	Eukarya	CBFB	UNKNOWN	Metazoa
ZBED1	UNKNOWN	Eukarya	CPEB1	UNKNOWN	Metazoa
EP300	UNKNOWN	Eukarya	LCOR	HTH_psq	post Metazoa (Bilateria)
CNOT3	UNKNOWN	Eukarya	PROX1	Prox1	post Metazoa (Bilateria)
FUBP1	UNKNOWN	Eukarya	GTF2IRD1	GTF2I	post Metazoa (Chordata)
CCNT2	UNKNOWN	Eukarya	SPZ1	UNKNOWN	post Metazoa (Eutheria)
GTF3C2	UNKNOWN	Eukarya	HMGN3	UNKNOWN	post Metazoa (Euteleostomi)
TRIM28	UNKNOWN	Eukarya	POU2AF1	UNKNOWN	post Metazoa (Euteleostomi)
TPARP1	UNKNOWN	Eukarya	BCLAF1	UNKNOWN	post Metazoa (Euteleostomi)
CEBPZ	UNKNOWN	Eukarya	NAIF1	MADF_DNA_bdg	post Metazoa (Euteleostomi)

8 List of connected components of the motif familes network

Families with size ≥ 10

famID245
DBD: **Fork_head** size: **41**
clique: **False** density: **0.74**
FOXS1 FOXN3 FOXD3 FOXE3 FOXA1 FOXD2 FOXQ1 AP5Z1 FOXP4 FOXK2 FOXC1 FOXL1 FOXB1 FOXB2 FOXD4L1 FOXK1 FOXF2 FOXJ2 FOXA3 FOXP2 FOXG1 FOXD4L3 FOXJ1 FOXI2 FOXE1 FOXD4L5 FOXC2 FOXP3 FOXD4 FOXD4L4 FOXD1 FOXL2 FOXN2 FOXM1 FOXI1 FOXJ3 FOXN1 FOXN4 FOXA2 FOXP1 FOXF1

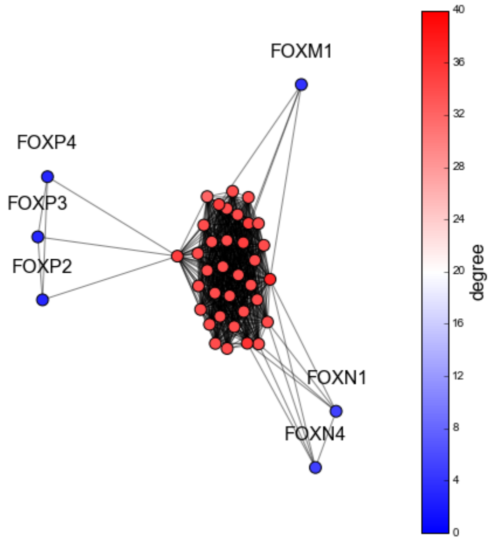

famID041
DBD: **Homeobox** size: **34**
clique: **False** density: **0.73**
HOXA5 HOXA6 HOXD9 HOXC11 HOXA2 HOXA3 HOXA1 HOXA11 HOXC10 HOXC5 HOXC8 GSX1 HOXB9 HOXB4 HOXD11 HOXC9 GSX2 PDX1 HOXB8 HOXB2 HOXD1 HOXD3 HOXD10 HOXD4 HOXD8 HOXC6 HOXB5 HOXA4 HOXB1 HOXB3 HOXA9 HOXA7 HOXC4 HOXB6

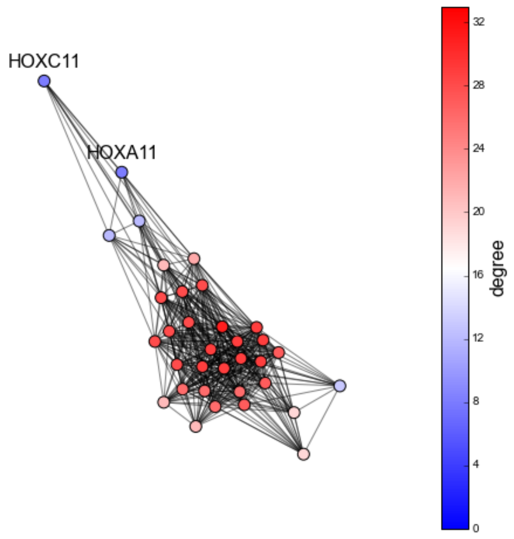

famID045
DBD: **Homeobox** size: **25**
clique: **False** density: **0.80**
VSX2 ALX3 VSX1 ALX4 PRRX1 PRRX2 ISX PROP1 ARX DMBX1 OTX1 PHOX2A RAX CRX OTX2 OTP PHOX2B UNCX DRGX SHOX GSC2 GSC ALX1 RAX2 SHOX2

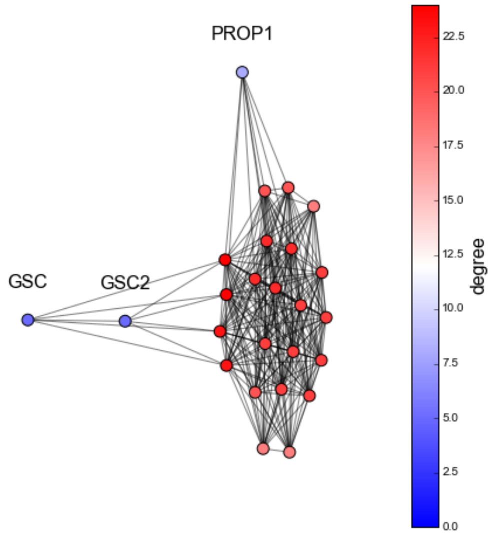

famID315
DBD: **Ets** size: **15**
clique: **True** density: **1.00**
ETS1 ETV5 ELK4 FLI1 ELK1 GABPA FEV ETV2 ERF ETS2 ERG ETV4 ETV3 ETV1 ELK3

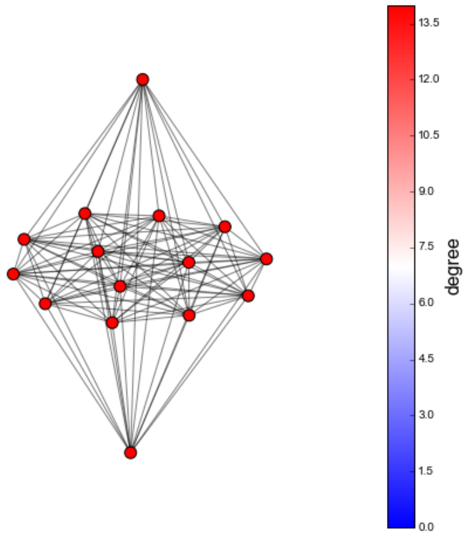

famID316
DBD: **Pou** size: **15**
clique: **False** density: **0.96**
POU2F1 POU3F2 POU5F1 POU3F4 POU5F1 POU5F1 POU5F1 POU3F3 POU5F1 POU5F1 POU2F2 POU3F1 POU5F1B POU5F1 POU2F3

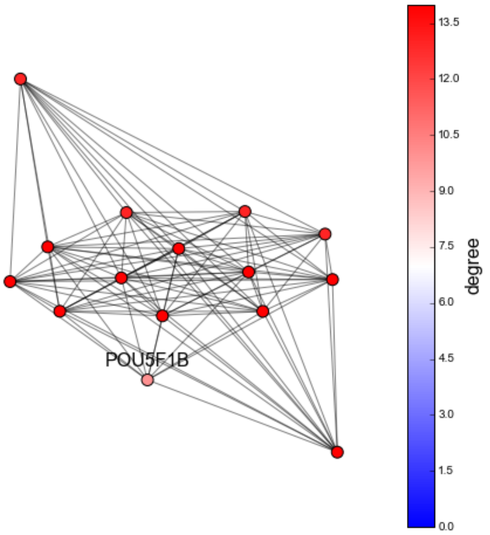

famID135
DBD: **zf-C2H2** size: **14**
clique: **False** density: **0.55**
SP5 KLF11 SP6 KLF9 SP8 SP1 SP4 SP7 KLF16 SP2 SP3 KLF13 KLF10 SP9

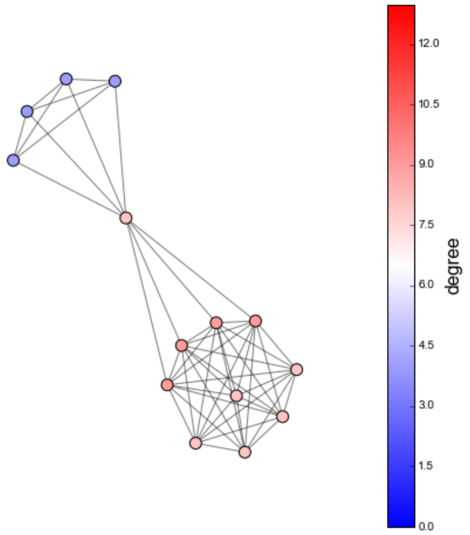

famID024
DBD: **Homeobox** size: **12**
clique: **False** density: **0.64**
NKX2-3 NKX2-2 HMX3 NKX3-2 NKX2-8 HMX2 NKX2-4 NKX3-1 NKX2-5 HMX1 NKX2-1 NKX2-6

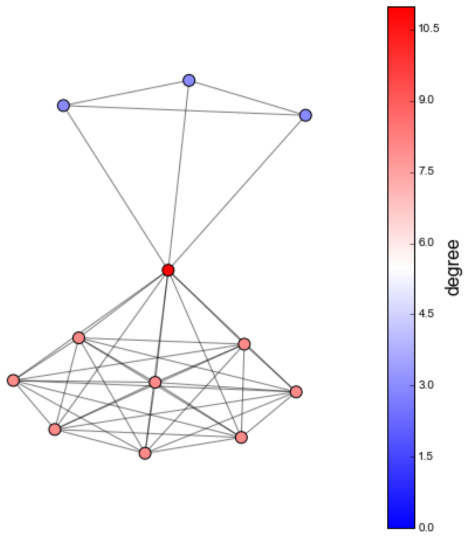

famID039
DBD: **Homeobox** size: **10**
clique: **True** density: **1.00**
PBX2 PBX3 PBX2 PBX2 PBX2 PBX2 PBX2 PBX1 PBX2 PBX4

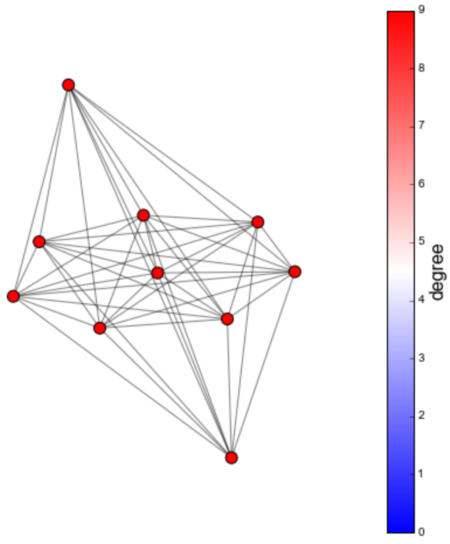

Families with size 9

famID091 DBD: **zf-C2H2**
clique: **True** density: **1.00**
KLF7 KLF6 KLF4 KLF2 KLF3 KLF5 KLF1 KLF8 KLF12

famID346 DBD: **RFX_DNA_binding**
clique: **True** density: **1.00**
RFX4 ARID2 RFX3 RFX5 RFX8 RFX2 RFX6 RFX7 RFX1

famID178 DBD: **HLH**
clique: **False** density: **0.89**
NEUROD2 NEUROG3 ATOH7 NEUROD4 NEUROG2 NEUROD6 NEUROG1 NEUROD1 ATOH1

Families with size 8

famID240 DBD: **zf-C4**
clique: **True** density: **1.00**
RXRB RXRA RXRB RXRG RXRB RXRB RXRB RXRB

Families with size 7

famID321 DBD: **T-box**
clique: **False** density: **0.62**
TBX2 TBX5 TBX20 TBX1 TBX4 TBX3 TBX10

Families with size 6

famID004 DBD: **Homeobox**
clique: **False** density: **0.80**
SIX3 SIX2 SIX5 SIX6 SIX1 SIX4

famID013 DBD: **Homeobox**
clique: **True** density: **1.00**
IRX2 IRX4 IRX1 IRX5 IRX6 IRX3

famID193 DBD: **HLH**
clique: **False** density: **0.87**
HIF1A SIM1 NPAS3 EPAS1 NPAS1 SIM2

famID374 DBD: **GATA**
clique: **True** density: **1.00**
GATA4 GATA3 GATA5 GATA2 GATA6 GATA1

famID011 DBD: **Homeobox**
clique: **True** density: **1.00**
DLX5 DLX6 DLX4 DLX1 DLX2 DLX3

famID056 DBD: **zf-C2H2**
clique: **True** density: **1.00**
ZNF705D ZNF705B ZNF705E ZNF705A ZNF705CP ZNF705G

famID197 DBD: **HLH**
clique: **False** density: **0.60**
TCF21 TWIST2 MSC TWIST1 TCF23 TCF15

Families with size 5

famID040 DBD: **Homeobox**
clique: **True** density: **1.00**
MEIS1 MEIS2 PKNOX1 MEIS3 PKNOX2

famID137 DBD: **zf-C2H2**
clique: **True** density: **1.00**
ZIC2 ZIC1 ZIC5 ZIC4 ZIC3

famID204 DBD: **HLH**
clique: **True** density: **1.00**
BHLHE23 OLIG1 OLIG2 OLIG3 BHLHE22

famID213 DBD: **bZIP_1**
clique: **False** density: **0.90**
CREB3L1 CREB3L3 CREB3L2 CREB3 CREB3L4

famID326 DBD: **MH1**
clique: **False** density: **0.80**
SMAD1 SMAD2 SMAD5 SMAD3 SMAD9

famID372 DBD: **SRF-TF**
clique: **True** density: **1.00**
MEF2C MEF2D BORCS8-MEF2B MEF2A MEF2B

famID064 DBD: **zf-C2H2**
clique: **True** density: **1.00**
ZBTB12 ZBTB12 ZBTB12 ZBTB12 ZBTB12

famID190 DBD: **HLH**
clique: **True** density: **1.00**
NHLH1 LYL1 NHLH2 TAL2 TAL1

famID205 DBD: **bZIP_1**
clique: **True** density: **1.00**
ATF6B ATF6B ATF6B ATF6 ATF6B

famID238 DBD: **zf-C4**
clique: **False** density: **0.70**
NR2F1 NR2C1 NR2F6 NR2C2 NR2F2

famID345 DBD: **PAX**
clique: **True** density: **1.00**
PAX9 PAX5 PAX1 PAX8 PAX2

famID388 DBD: **TF_AP-2**
clique: **True** density: **1.00**
TFAP2B TFAP2D TFAP2E TFAP2A TFAP2C

Families with size 4

famID000 DBD: **Homeobox**
clique: **True** density: **1.00**
HOXC13 HOXA13 HOXD13 HOXB13

famID073 DBD: **zf-C2H2**
clique: **True** density: **1.00**
EGR2 EGR4 EGR1 EGR3

famID030 DBD: **Homeobox**
clique: **True** density: **1.00**
TGIF2LY TGIF2LX TGIF2 TGIF1

famID175 DBD: **HLH**
clique: **True** density: **1.00**
MYOD1 MYF6 MYF5 MYOG

famID185 DBD: **HLH**
clique: **True** density: **1.00**
ARNTL ARNT ARNT2 ARNTL2

famID189 DBD: **HLH**
clique: **True** density: **1.00**
TFE3 TFEB MITF TFEC

famID198 DBD: **HLH**
clique: **True** density: **1.00**
ID2 ID3 ID1 ID4

famID212 DBD: **bZIP_1**
clique: **True** density: **1.00**
CEBPA CEBPD CEBPB CEBPE

famID217 DBD: **bZIP_1**
clique: **True** density: **1.00**
MAFA MAF MAFB NRL

famID218 DBD: **bZIP_1**
clique: **False** density: **0.83**
NFE2L3 NFE2L1 NFE2L2 NFE2

famID224 DBD: **bZIP_1**
clique: **True** density: **1.00**
FOSB FOSL2 FOS FOSL1

famID230 DBD: **zf-C4**
clique: **True** density: **1.00**
PGR NR3C2 AR NR3C1

famID246 DBD: **Fork_head**
clique: **True** density: **1.00**
FOXO4 FOXO1 FOXO3 FOXO6

famID304 DBD: **HMG_box**
clique: **True** density: **1.00**
SOX1 SOX14 SOX21 SOX2

famID308 DBD: **HMG_box**
clique: **True** density: **1.00**
LEF1 TCF7 TCF7L2 TCF7L1

famID329 DBD: **MH1**
clique: **True** density: **1.00**
NFIB NFIC NFIA NFIX

famID337 DBD: **RHD**
clique: **True** density: **1.00**
NFATC3 NFATC2 NFATC1 NFATC4

Families with size 3

famID001 DBD: **Homeobox**
clique: **True** density: **1.00**
NKX6-1 NKX6-3 NKX6-2

famID007 DBD: **Homeobox**
clique: **True** density: **1.00**
TLX1 TLX3 TLX2

famID019 DBD: **Homeobox**
clique: **False** density: **0.67**
BARX1 BARX2 BSX

famID021 DBD: **Homeobox**
clique: **True** density: **1.00**
GBX2 GBX1 MNX1

famID025 DBD: **Homeobox**
clique: **True** density: **1.00**
LHX5 LHX3 LHX4

famID028 DBD: **Homeobox**
clique: **True** density: **1.00**
CDX1 CDX2 CDX4

famID048 DBD: **Homeobox**
clique: **True** density: **1.00**
PITX2 PITX1 PITX3

famID055 DBD: **zf-C2H2**
clique: **True** density: **1.00**
ZFP42 YY2 YY1

famID086 DBD: **zf-C2H2**
clique: **False** density: **0.67**
SALL1 SALL4 SALL3

famID102 DBD: **zf-C2H2**
clique: **True** density: **1.00**
HIVEP3 HIVEP1 HIVEP2

famID152 DBD: **zf-C2H2**
clique: **True** density: **1.00**
GLI2 GLI1 GLI3

famID174 DBD: **HLH**
clique: **True** density: **1.00**
TCF3 TCF12 TCF4

famID177 DBD: **HLH**
clique: **True** density: **1.00**
HEY1 HEYL HEY2

famID181 DBD: **HLH**
clique: **True** density: **1.00**
HES1 HES4 HES2

famID206 DBD: **bZIP_1**
clique: **True** density: **1.00**
CREB1 ATF1 CREM

famID207 DBD: **bZIP_1**
clique: **True** density: **1.00**
JUND JUN JUNB

famID208 DBD: **bZIP_1**
clique: **True** density: **1.00**
DBP HLF TEF

famID216 DBD: **bZIP_1**
clique: **True** density: **1.00**
MAFG MAFF MAFK

famID221 DBD: **bZIP_1**
clique: **True** density: **1.00**
CREB5 ATF7 ATF2

famID227 DBD: **zf-C4**
clique: **False** density: **0.67**
NR1H3 NR1H4 NR1H2

famID229 DBD: **zf-C4**
clique: **True** density: **1.00**
NR4A2 NR4A3 NR4A1

famID233 DBD: **zf-C4**
clique: **True** density: **1.00**
RORB RORC RORA

famID235 DBD: **zf-C4**
clique: **True** density: **1.00**
RARA RARG RARB

famID241 DBD: **zf-C4**
clique: **True** density: **1.00**
PPARD PPARA PPARG

famID242 DBD: **zf-C4**
clique: **True** density: **1.00**
ESRRG ESRRB ESRRA

famID301 DBD: **HMG_box**
clique: **True** density: **1.00**
SOX10 SOX9 SOX8

famID303 DBD: **HMG_box**
clique: **True** density: **1.00**
SOX13 SOX5 SOX6

famID310 DBD: **Ets**
clique: **True** density: **1.00**
CTD-2545M3.6 SPIC SPI1

famID313 DBD: **Ets**
clique: **True** density: **1.00**
ELF5 EHF ELF3

famID314 DBD: **Ets**
clique: **True** density: **1.00**
ELF2 ELF1 ELF4

famID319 DBD: **Pou**
clique: **True** density: **1.00**
POU4F1 POU4F3 POU4F2

famID320 DBD: **T-box**
clique: **True** density: **1.00**
TBX18 TBX22 TBX15

famID324 DBD: **T-box**
clique: **True** density: **1.00**
TBR1 TBX21 EOMES

famID331 DBD: **E2F_TDP**
clique: **True** density: **1.00**
TFDP3 TFDP1 TFDP2

famID340 DBD: **RHD**
clique: **False** density: **0.67**
REL RELA NFKB1

famID370 DBD: **DM**
clique: **False** density: **0.67**
DMRT3 DMRT2 DMRTA2

famID377 DBD: **CUT**
clique: **True** density: **1.00**
ONECUT3 ONECUT2 ONECUT1

famID382 DBD: **ARID**
clique: **True** density: **1.00**
ARID3A ARID3C ARID3B

famID403 DBD: **Runt**
clique: **True** density: **1.00**
RUNX3 RUNX1 RUNX2

famID404 DBD: **CSD**
clique: **True** density: **1.00**
YBX1 YBX3 YBX2

Families with size 2

famID002 DBD: **Homeobox**
clique: **True** density: **1.00**
LMX1A LMX1B

famID005 DBD: **Homeobox**
clique: **True** density: **1.00**
EMX2 EMX1

famID008 DBD: **Homeobox**
clique: **True** density: **1.00**
LHX9 LHX2

famID009 DBD: **Homeobox**
clique: **True** density: **1.00**
LHX6 LHX8

famID016 DBD: **Homeobox**
clique: **True** density: **1.00**
DBX2 HLX

famID022 DBD: **Homeobox**
clique: **True** density: **1.00**
LBX2 LBX1

famID023 DBD: **Homeobox**
clique: **True** density: **1.00**
EVX2 EVX1

famID029 DBD: **Homeobox**
clique: **True** density: **1.00**
VAX2 VAX1

famID031 DBD: **Homeobox**
clique: **True** density: **1.00**
MSX1 MSX2

famID032 DBD: **Homeobox**
clique: **True** density: **1.00**
NKX1-1 NKX1-2

famID036 DBD: **Homeobox**
clique: **True** density: **1.00**
EN1 EN2

famID037 DBD: **Homeobox**
clique: **True** density: **1.00**
ISL1 ISL2

famID038 DBD: **Homeobox**
clique: **True** density: **1.00**
BARHL2 BARHL1

famID042 DBD: **Homeobox**
clique: **True** density: **1.00**
MEOX2 MEOX1

famID049 DBD: **Homeobox**
clique: **True** density: **1.00**
HOXC12 HOXD12

famID051 DBD: **Homeobox**
clique: **True** density: **1.00**
NANOGP1 NANOG

famID057 DBD: **zf-C2H2**
clique: **True** density: **1.00**
ZBTB7A ZBTB7C

famID059 DBD: **zf-C2H2**
clique: **True** density: **1.00**
ZNF148 ZNF281

famID065 DBD: **zf-C2H2**
clique: **True** density: **1.00**
MECOM PRDM16

famID066 DBD: **zf-C2H2**
clique: **True** density: **1.00**
FEZF2 FEZF1

famID069 DBD: **zf-C2H2**
clique: **True** density: **1.00**
ZBTB47 ZNF652

famID070 DBD: **zf-C2H2**
clique: **True** density: **1.00**
BCL11A BCL11B

famID071 DBD: **zf-C2H2**
clique: **True** density: **1.00**
IKZF3 IKZF2

famID074 DBD: **zf-C2H2**
clique: **True** density: **1.00**
ZFY ZFX

famID075 DBD: **zf-C2H2**
clique: **True** density: **1.00**
HIC1 HIC2

famID081 DBD: **zf-C2H2**
clique: **True** density: **1.00**
SNAI1 SNAI3

famID089 DBD: **zf-C2H2**
clique: **True** density: **1.00**
PRDM1 ZNF683

famID094 DBD: **zf-C2H2**
clique: **True** density: **1.00**
ZNF143 ZNF76

famID099 DBD: **zf-C2H2**
clique: **True** density: **1.00**
OSR2 OSR1

famID118 DBD: **zf-C2H2**
clique: **True** density: **1.00**
GFI1 GFI1B

famID140 DBD: **zf-C2H2**
clique: **True** density: **1.00**
IKZF1 IKZF4

famID144 DBD: **zf-C2H2**
clique: **True** density: **1.00**
ZNF75D ZNF75A

famID148 DBD: **zf-C2H2**
clique: **True** density: **1.00**
ZKSCAN3 ZKSCAN4

famID158 DBD: **zf-C2H2**
clique: **True** density: **1.00**
ZBTB42 ZBTB18

famID168 DBD: **HLH**
clique: **True** density: **1.00**
MYCL MYCN

famID171 DBD: **HLH**
clique: **True** density: **1.00**
SREBF1 SREBF2

famID172 DBD: **HLH**
clique: **True** density: **1.00**
USF2 USF1

famID173 DBD: **HLH**
clique: **True** density: **1.00**
CLOCK NPAS2

famID180 DBD: **HLH**
clique: **True** density: **1.00**
MESP2 MESP1

famID182 DBD: **HLH**
clique: **True** density: **1.00**
HAND2 HAND1

famID183 DBD: **HLH**
clique: **True** density: **1.00**
PTF1A FERD3L

famID187 DBD: **HLH**
clique: **True** density: **1.00**
AHRR AHR

famID188 DBD: **HLH**
clique: **True** density: **1.00**
BHLHE41 BHLHE40

famID195 DBD: **HLH**
clique: **True** density: **1.00**
ASCL2 ASCL1

famID200 DBD: **HLH**
clique: **True** density: **1.00**
CCDC169-SOHLH2 SOHLH2

famID209 DBD: **bZIP_1**
clique: **True** density: **1.00**
BACH1 BACH2

famID211 DBD: **bZIP_1**
clique: **True** density: **1.00**
ATF4 ATF5

famID223 DBD: **bZIP_1**
clique: **True** density: **1.00**
JDP2 ATF3

famID226 DBD: **zf-C4**
clique: **True** density: **1.00**
NR5A2 NR5A1

famID228 DBD: **zf-C4**
clique: **True** density: **1.00**
NR1D2 NR1D1

famID237 DBD: **zf-C4**
clique: **True** density: **1.00**
HNF4G HNF4A

famID244 DBD: **zf-C4**
clique: **True** density: **1.00**
ESR2 ESR1

famID298 DBD: **HMG_box**
clique: **True** density: **1.00**
SOX11 SOX4

famID317 DBD: **Pou**
clique: **True** density: **1.00**
POU6F2 POU6F1

famID332 DBD: **E2F_TDP**
clique: **True** density: **1.00**
E2F4 E2F5

famID344 DBD: **PAX**
clique: **True** density: **1.00**
PAX3 PAX7

famID358 DBD: **Myb_DNA-binding**
clique: **True** density: **1.00**
MYB MYBL1

famID375 DBD: **CUT**
clique: **True** density: **1.00**
SATB1 SATB2

famID380 DBD: **HSF_DNA-bind**
clique: **True** density: **1.00**
HSF1 HSF4

famID398 DBD: **CP2**
clique: **True** density: **1.00**
UBP1 TFCP2L1

famID407 DBD: **AT_hook**
clique: **True** density: **1.00**
HMGA1 HMGA2

famID411 DBD: **TBP**
clique: **True** density: **1.00**
TBPL2 TBP

famID243 DBD: **zf-C4**
clique: **True** density: **1.00**
THRA THRB

famID248 DBD: **Fork_head**
clique: **True** density: **1.00**
FOXR2 FOXR1

famID311 DBD: **Ets**
clique: **True** density: **1.00**
ETV6 ETV7

famID323 DBD: **T-box**
clique: **True** density: **1.00**
TBX19 T

famID334 DBD: **E2F_TDP**
clique: **True** density: **1.00**
E2F3 E2F2

famID357 DBD: **Myb_DNA-binding**
clique: **True** density: **1.00**
SMARCC1 SMARCC2

famID365 DBD: **STAT_bind**
clique: **True** density: **1.00**
STAT5A STAT5B

famID379 DBD: **HSF_DNA-bind**
clique: **True** density: **1.00**
HSFY1 HSFY2

famID381 DBD: **ARID**
clique: **True** density: **1.00**
ARID5A ARID5B

famID405 DBD: **P53**
clique: **True** density: **1.00**
TP63 TP73

famID410 DBD: **GCM**
clique: **True** density: **1.00**
GCM1 GCM2

Families with size 1

DBD **zf-C2H2** ZNF628 ZNF713 E4F1 ZNF182 IKZF5 ZNF274 OVOL2 ZNF219 ZNF664 ZNF615 INSM1 ZNF384 ZNF658B ZNF521 TRERF1 ZNF410 PLAG1 ZNF761 ZNF589 GLIS2 ZBTB4 ZNF718 PLAGL1 ZBTB3 ZEB1 RREB1 ZSCAN10 SCRT2 ZBTB14 SALL2 ZSCAN26 GLIS1 MTF1 ZSCAN16 ZNF32 ZNF354C ZNF217 ZBTB7B ZNF679 BCL6B ZBTB6 ZNF524 ZBTB16 BCL6 ZNF423 ZNF232 REST ZNF425 PATZ1 HINFP CTCFL ZNF541 ZNF784 ZKSCAN1 GLIS3 ZNF333 ZNF581 ZNF202 ZNF282 MZF1 PRDM15 ZNF691 CTCF ZBTB49 PRDM12 ZBTB1 ZBTB11 WT1 MAZ ZNF316 ZNF697 SNAI2 GTF3A ZNF90 ZNF740 ZBTB33 ZNF467 PRDM4 ZNF263 KLF15 ZFP1 ZSCAN4 ZNF35 OVOL1 GZF1 PLAGL2 ZNF236

DBD **UNKNOWN** TAF1 BPTF BRF1 LMO2 ZBED1 GABPB1 ENO1 EP300 NRF1 EBF4 CBFB PURA SPZ1 HDAC2 CNOT3 FUBP1 CCNT2 GTF3C2 NFYB BCL3 HMGN3 CHD2 CPEB1 SIRT6 HLTF EBF2 TRIM28 PARP1 CTNNB1 POU2AF1 GTF2B SMC3 CEBPZ SIN3A ZNF350 POLR3A LTF NFYC BCLAF1 TOPORS HDAC1 RAD21 NR0B1 EBF3

DBD **Homeobox** HMBOX1 VENTX VTN HNF1A ZHX1 ZFHX3 RHOXF1 DPRX ZFHX2 ANHX HDX HESX1 ESX1 NOBOX HOMEZ NOTO DUX4 HHEX MIXL1

DBD **HLH** HES5 MYC MLX NPAS4 MLXIPL MXI1 EBF1 ATOH8 BHLHA15 MNT MLXIP FIGLA TFAP4 HES7 TCFL5

DBD **HMG_box** BBX SOX15 SOX17 HBP1 HMG20B CIC SOX18 SOX3 SOX30 SOX7 SOX12 SRY

DBD **IRF** IRF3 IRF7 IRF6 IRF5 IRF8 IRF2 IRF1 IRF9 IRF4

DBD **bZIP_1** CEBPG XBP1 NFIL3 BATF MAX DDIT3 BATF3

DBD **zf-CXXC** TET1 CXXC1 KDM2B DNMT1 KMT2A

DBD **zf-C4** NR2E1 NR1I3 VDR NR6A1 NR1I2

DBD **STAT_bind** STAT1 STAT4 STAT3 STAT6 STAT2

DBD **E2F_TDP** E2F7 E2F8 E2F1 E2F6

DBD **TEA** TEAD1 TEAD2 TEAD3 TEAD4

DBD **SAND** GMEB1 GMEB2 AIRE DEAF1

DBD **Myb_DNA-binding** MYBL2 CDC5L TERF2 MYPOP

DBD **DM** DMRTC2 DMRT1 DMRTA1

DBD **MBD** MECP2 MBD2 SETDB1

DBD **MH1** SMAD4 SMAD6 SMAD7

DBD **RHD** NFKB2 RELB NFAT5

DBD **PAX** PAX6 PAX4

DBD **THAP** PRKRIR THAP1

DBD **CP2** GRHL1 TFCP2

DBD **NDT80_PhoG** MYRF

DBD **GAGA_bind** ING4

DBD **HSF_DNA-bind** HSF2

DBD **mTERF** MTERF1

DBD **SRF-TF** SRF

DBD **LAG1-DNAbind** RBPJ

DBD **GTF2I** GTF2IRD1

DBD **MADF_DNA_bdg** NAIF1

DBD **Ets** SPDEF

DBD **HTH_psq** LCOR

DBD **CBFB_NFYA** NFYA

DBD **CUT** CUX2

DBD **EIN3** BRCA1

DBD **Fork_head** FOXH1

DBD **T-box** MGA

DBD **Pou** POU1F1

DBD **Prox1** PROX1

DBD **CENP-B_N** CENPB

DBD **CXC** LIN54

DBD **P53** TP53
